# Enhancing Colistin Activity against Colistin-Resistant *Escherichia coli* through Combination with Alginate Nanoparticles and Small Molecules

**DOI:** 10.3390/ph15060682

**Published:** 2022-05-28

**Authors:** Noura Hazime, Yanath Belguesmia, Isabelle Kempf, Alexandre Barras, Djamel Drider, Rabah Boukherroub

**Affiliations:** 1 Univ. Lille, CNRS, Centrale Lille, Univ. Polytechnique Hauts-de-France, UMR 8520, IEMN, F-59000 Lille, France; noura.hazime@hotmail.fr (N.H.); alexandre.barras@univ-lille.fr (A.B.); 2 UMR Transfrontalière BioEcoAgro1158, Univ. Lille, INRAE, Univ. Liège, UPJV, YNCREA, Univ. Artois, Univ. Littoral Côte D’Opale, ICV-Institut Charles Viollette, 59000 Lille, France; yanath.belguesmia@univ-lille.fr (Y.B.); djamel.drider@univ-lille.fr (D.D.); 3 Agence Nationale de Sécurité Sanitaire de L'Alimentation, de L'Environnement et du Travail, Laboratoire de Ploufragan-Plouzané-Niort, Unité Mycoplasmologie Bactériologie Antibiorésistance, 22440 Ploufragan, France; isabelle.kempf@anses.fr

**Keywords:** alginate nanoparticles, colistin, essential oils, polyamines, lactic acid, *Escherichia coli*, antimicrobial activity

## Abstract

Bacterial resistance to antibiotics has become a major public health problem worldwide, with the yearly number of deaths exceeding 700,000. To face this well-acknowledged threat, new molecules and therapeutic methods are considered. In this context, the application of nanotechnology to fight bacterial infection represents a viable approach and has experienced tremendous developments in the last decades. *Escherichia coli* (*E. coli*) is responsible for severe diarrhea, notably in the breeding sector, and especially in pig farming. The resulting infection (named colibacillosis) occurs in young piglets and could lead to important economic losses. Here, we report the design of several new formulations based on colistin loaded on alginate nanoparticles (Alg NPs) in the absence, but also in the presence, of small molecules, such as components of essential oils, polyamines, and lactic acid. These new formulations, which are made by concomitantly binding colistin and small molecules to Alg NPs, were successfully tested against *E. coli* 184, a strain resistant to colistin. When colistin was associated with Alg NPs, the minimal inhibition concentration (MIC) decreased from 8 to 1 µg/mL. It is notable that when menthol or lactic acid was co-loaded with colistin on Alg NPs, the MIC of colistin drastically decreased, reaching 0.31 or 0.62 µg/mL, respectively. These novel bactericidal formulations, whose innocuity towards eukaryotic HT-29 cells was established in vitro, are presumed to permeabilize the bacterial membrane and provoke the leakage of intracellular proteins. Our findings revealed the potentiating effect of the Alg NPs on colistin, but also of the small molecules mentioned above. Such ecological and economical formulations are easy to produce and could be proposed, after confirmation by in vivo and toxicology tests, as therapeutic strategies to replace fading antibiotics.

## 1. Introduction

Nanotechnology has emerged as a powerful tool in the biomedical and pharmaceutical fields to treat antibiotic-resistant strains, especially Gram-negative bacteria [1], among which *Escherichia coli* (*E. coli*) is considered the most common bacterial pathogen responsible for diarrheal diseases [2]. Antibacterial compounds, such as antibiotics, can be classified into bactericidal and bacteriostatic agents, which respectively kill the target bacteria or slow down its growth. The overuse of antibiotics has contributed to the emergence of bacterial resistance, which currently represents a major public health concern [3]; this situation is aggravated by the lack of new antibiotics on the market.

To face this alarming situation, several strategies have been considered, including nanosystems, which have the ability to deliver antibiotics and make them more bioavailable at infection sites [4]. In addition, nanoparticle-based delivery systems have the potential to improve drug stability, increase the duration of the therapeutic effect, and allow administration through enteral or parenteral pathways, which may prevent or minimize drug degradation and metabolism as well as cellular efflux [5,6]. Notably, several classes of antimicrobial nanoparticles and nanosized carriers for antibiotics’ delivery have proven their effectiveness for treating infectious diseases, including those due to antibiotic-resistant bacteria [7].

Colistin is an antibiotic of the polymyxin family that is active against Gram-negative bacteria. This family of antibiotics consists of five members, that is, polymyxins A to E [8]. However, only polymyxins B and E are used in human and/or veterinary medicine. It should be noted that colistin was withdrawn from human medical practice due to its side effects, such as renal toxicity [9], before being reintroduced recently as a drug of last resort to treat severe infection caused by multi-drug-resistant bacteria [10]. Importantly, colistin has remained in the veterinary therapeutic arsenal to treat infections caused by Gram-negative bacilli [11]. Another argument to support the therapeutic use of colistin was related to the low level of resistance recorded up to the beginning of the 2010s, but also to the apparent absence of transferable resistance mechanisms through mobile genetic elements (MGE), such as plasmids. Nonetheless, in 2015, the situation regarding colistin resistance changed with the discovery of *E. coli* strains carrying plasmid genetic resistance, known as the *mcr*-1 gene [12]. To date, several genes (named *mcr*-1 to *mcr*-10) have been discovered throughout the world [13,14], indicating potential dissemination of a colistin resistance trait, rendering complicated the treatment of humans and animals affected by infections caused by these pathogens [13,15].

The development of nanoparticles from natural and biodegradable polymers to deliver drugs, in order to replace metallic ones, has gained substantial interest. Related to this, alginate is a very promising support, widely considered in the pharmaceutical field for controlling drug release [16]. Thus, alginate has been extensively reviewed for its physical and chemical properties and use in the formation of (micro) particles and bulk gels [17,18,19]. Interestingly, alginate is non-toxic, biodegradable, low cost, readily available, and has been found to be a mucoadhesive, biocompatible, and non-immunogenic substance. This anionic polymer, produced by brown algae and bacteria, consists of α-l-glucuronic acid (G) and β-d-mannuronic acid (M) residues linearly linked by 1, 4-glycosidic units. It is notable that alginate can also be chemically modified to adapt its properties to the targeted application [20,21].

Previous studies established the synergistic activities of nanoparticles and essential oils against various drug-resistant microorganisms [22,23,24]. The present study aims to evaluate the potentializing effect of alginate nanoparticles (Alg NPs) as a nanocarrier of colistin, as well as small molecules, such as components of essential oils, polyamines, and lactic acid. The antimicrobial activities of these formulations and their cytotoxicity were evaluated in vitro to explore their therapeutic efficacy, while reducing their undesirable effects. These “nanometric carriers” are able to break through barriers, protect the transported molecules, and deliver them in biologically-active form to the site of action.

## 2. Results and Discussion

### 2.1. Combination of Colistin with Alg NPs Has Increased Antibacterial Activity

Notably, the size of the obtained Alg NPs ranged from 72 to 130 nm, with an average of 111 nm (data not shown). A formulation of Alg NPs-Colistin (500:60 µg/mL) was tested against the *E. coli* strains listed in Table 1. The antimicrobial activity, determined by MIC values, is given in Table 1, which delineates two groups. The first group is composed of *E. coli* strains 184 and 289, resistant to colistin according to EUCAST and CLSI recommendations [25,26], and the second group comprises the remaining strains, which are sensitive to colistin.

Importantly, upon treatment of *E. coli* strains 184 and 289 with the Alg NPs+Colistin formulation (500:60 µg/mL), their MIC values decreased by at least four-fold. Despite the decrease of its MIC, the later strain remained resistant to colistin [25,26]. On the other hand, treatment of *E. coli* ATCC 8739, SBS363, and Top 10 strains with the Alg NPs+Colistin formulation constantly reduced the MIC values by at least two-fold in each of the three repeated assays made.

Colistin is a bactericidal antibiotic directed against Gram-negative bacteria by targeting their lipopolysaccharide (LPS) [27,28], causing a permeability of their cytoplasmic membrane. The resistance to colistin has been associated with modifications occurring on the LPS by lowering the affinity of colistin to LPS. Resistance to colistin of *E. coli* 184 is mediated by the *mcr*-1 gene, which was reported for the first time in 2016 in *Enterobacterales* [12]. According to PCR, the *E. coli* 289 strain carries the *mcr*-4 gene [29], but we cannot exclude the presence of other resistance mechanisms, such as mutations in target genes (e.g., *mgrB, phoP, phoQ*, *pmrB*) [30]. Usually, strains carrying the *mcr*-1 or *mcr*-4 resistance feature a low level of resistance, with MIC values ranging from 4 to 8 µg/mL [30]. The combination of colistin with Alg NPs enhanced its antibacterial activity as the MIC value of the antibiotic diminished, delineating a potentiating effect of Alg NPs. Similar potentiating effects have been reported for azithromycin or erythromycin combined with alginate G oligosaccharides (OligoG) [31]. Similarly, Pritchard et al. showed that the combination of colistin with low-molecular-weight alginate oligoG disrupted pseudomonal micro colony formation and improved the efficacy of colistin in reducing pseudomonal biofilm biomass [32].

### 2.2. Combination of Colistin with Alg NPs and Components of Essential Oils

Several works demonstrated the antimicrobial effect of components of essential oils on a wide range of Gram-positive and Gram-negative bacteria [33]. Even though components of essential oils exhibit a broader spectrum of activity, they display lower inhibition efficiency than antibiotics. In our study, we considered menthol, carvacrol, geraniol, and farnesol. Components of essential oils were combined with Alg NPs to gather novel formulations endowed with antibacterial activity. Here, we show that the Alg NPs+Menthol (500:10 µg/mL) formulation was not active against *E. coli* 184 under our experimental conditions. Nevertheless, when colistin was incorporated as a third component, the formulation became active against *E. coli* 184, with a MIC value varying from 0.31 to 1 µg/mL (Table 2), suggesting a potentiating effect of menthol with colistin and Alg NPs. Furthermore, it has been reported that menthol has antibacterial and antifungal activities, and the mode of action of menthol may result from a disruption of the lipid content of the bacterial membrane [34]. Moreover, menthol has shown synergistic interactions with antibiotics such as oxytetracycline, oxacillin, norfloxacin, or erythromycin [34,35].

To explore further how to recover the efficiency of colistin against resistant strains, we designed formulations comprising various components of essential oils, such as carvacrol, geraniol, and farnesol, based on the previously reported data [36,37]. When these components of essential oils were tested at 10 µg/mL in combination with Alg NPs, no antibacterial activity was registered against *E. coli* 184. Similarly, the incorporation of any of these components of essential oils at 10 µg/mL with colistin and Alg NPs resulted in formulations endowed with antibacterial activities. The most active formulation was Alg NPs+Colistin+Carvacrol, with a MIC value below 0.62 µg/mL (Table 2). Carvacrol is a monoterpene phenol, and isomer of thymol, exerting a bactericidal activity through alteration of the bacterial membrane [38]. According to Xu et al., carvacrol and thymol have an antibacterial effect on *E. coli* [39]. These antibacterial effects were attributed to their ability to permeabilize and depolarize the cytoplasmic membrane due to its hydrophobic structure. Components with a phenolic hydroxyl structure, such as eugenol and thymol, display antimicrobial activities against Gram-positive and Gram-negative bacteria, and also against yeasts. Interestingly, eugenol has been shown to synergistically interact with colistin and enhance its antimicrobial activity against colistin-resistant *E. coli* strains [40].

Furthermore, different studies reported that geraniol significantly increased the efficacy of β-lactams, quinolones, and chloramphenicol [41]. According to Lorenzi et al., geraniol significantly restores the susceptibility of the multi-drug-resistant *Enterobacter aerogenes* EAEP289 strain [41]. Taking into consideration all of these data, we postulate that geraniol is an essential oil component with an elevated therapeutic potential, and it could be used under certain conditions in the treatment of infectious diseases, at a time when antibiotics discovery is becoming scarce.

Similarly, farnesol has proven to be an antimicrobial agent by inhibiting the growth of pathogenic bacteria such as *Staphylococcus aureus* [42] and *Streptococcus mutans* [43]. According to Brehm et al., farnesol has the ability to increase bacterial permeability and susceptibility to a number of antibiotics [44]. Thus, farnesol was able to restore the sensitization of *E. coli* strains to polymyxin [44]. Chi et al. demonstrated the synergistic interactions between farnesol and polymyxin B against Gram-positive bacteria, such as *S. aureus* LMGT 3242 [45]. Another study found that farnesol could be a good adjuvant for colistin on *Acinetobacter baumannii* [46]. This study also revealed that combining colistin with farnesol increases the sensitivity of colistin-resistant strains to colistin. Farnesol may increase the activity of colistin by disrupting the outer membrane and thus increasing the ability of colistin to bind and solubilize membrane lipids [44,46].

Thus, essential oils are natural products with low costs, which are able to inhibit antibiotic-resistant pathogens or potentiate antibiotics, arguing for a possible smart usage during the well-documented antibiotic crisis.

### 2.3. Combination of Colistin with Alg NPs and Lactic Acid

The combination of Alg NPs with lactic acid (15 µg/mL) alone was not effective in inhibiting bacterial growth. In contrast, the MIC values of the Alg NPs+Colistin+Lactic acid formulation was reduced two-fold in comparison with Alg NPs+Colistin without lactic acid, on the *E. coli* 184 strain. This indicates that the combination of lactic acid also potentiates the activity of Alg NPs+Colistin (Table 3).

Organic acids are commonly applied in foods as preservatives to enhance their microbiological safety [47]. Organic acids were used in the bovine meat product processing industry to reduce bacterial contamination [48]. In addition, studies demonstrated that organic acids (acetic, citric, lactic, malic, propionic, and tartaric acids, among others) represent a potential alternative for controlling microbial contamination due to Gram-negative bacteria [49,50]. On the basis of the findings of Kashket et al., the inhibition of microbial growth by organic acids is ascribed to the ability of these acids to cross the cell membrane, dissociate internally at more alkaline pH, and acidify the cell cytoplasm [51]. Thus, organic acids may represent effective therapeutic alternatives for treating certain bacterial infections.

### 2.4. Combination of Colistin with Alg NPs and Polyamines (Spermine, Spermidine, Piperazine)

The antibacterial activity of the Alg NPs+Colistin+Spermidine (ACSd) formulation had the lowest MIC (2.5 µg/mL), and the Alg NPs+Colistin+Piperazine (ACP) formulation displayed the highest MIC (10 µg/mL) against *E. coli* 184 strain. The MIC of the ACP solution was four times higher than that of the ACSd solution (2.5 µg/mL). Regarding the activity of Alg NPs+Colistin+Spermine (ACS), the recorded MIC of 5 µg/mL was two times higher than that of the ACSd solution. Despite their structural similarities, spermine, which is a derivative of spermidine through the action of spermine synthetase, exhibited lower activity (Table 4).

Spermine has antibacterial activity against a wide range of Gram-positive and Gram-negative bacteria. The combination of polymyxin B with spermine decreases its MIC for *E. coli* K12 and *S. aureus* N315 strains by two folds (from 0.5 to 0.25 µg/mL) [52].

As the Alg NPs+Colistin+Spermidine (500:60:20 µg/mL) formulation designed in our study had the lowest MIC, we tested modulated concentrations of colistin and spermidine (i.e., ACSd_1_ (500:30:30 µg/mL), ACSd_2_ (500:20:40 µg/mL), and ACSd_3_ (500:10:50 µg/mL)). The results revealed that ACSd_1_ had the lowest MIC (3.75 µg/mL) compared to the other two formulations tested, ACSd_2_ and ACSd_3_, which displayed the same MIC values (5 µg/mL), despite the higher spermidine concentration. However, this activity was lower than that of the initial ACSd formulation, which had the higher colistin concentration (Table 4). Even if polyamines potentiate relatively the activity of Alg NPs+Colistin, the antibacterial activity remains linked to the colistin quantity in the formulation.

Natural polyamines, including cadaverine, putrescine, spermidine, and spermine, are a group of ubiquitous cationic compounds found in all living organisms. Spermine is present in eukaryotic cells, while the others are found in both prokaryotic and eukaryotic cells. Previous studies demonstrated the ability of spermine and spermidine to block solute diffusion in *E. coli* and to act as endogenous modulators of bacterial outer membrane permeability [53], inducing resistance to cationic peptide antibiotics, aminoglycosides, or quinolones [54]. On the contrary, and surprisingly, synthetic polyamine analogues have been reported to increase bacterial outer membrane permeability by disrupting the integrity of LPS, resulting in increased sensitivity of *E. coli* to hydrophobic antibiotics [53]. In addition, a series of chloramphenicol amides (CAMs) with polyamines (spermidine) have recently been synthesized and are among the most potent antibacterial agents against Gram-positive (*S. aureus*) and Gram-negative (*E. coli*) bacterial strains [55,56].

Thus, polyamine molecules could constitute an effective therapeutic alternative, acting on membrane depolarization and/or disrupting membrane integrity, and could considerably reduce the development of bacterial resistance.

### 2.5. Mode of Action of the Alg NPs+Colistin Formulation

**Figure 1:**
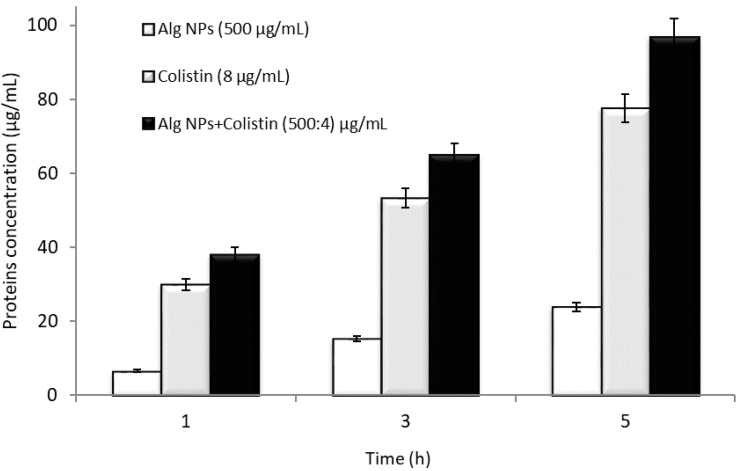
*Escherichia coli* 184 intracellular proteins leakage after treatments with colistin (8 µg/mL), Alg NPs (500 µg/mL), and Alg NPs+Colistin (500:60 µg/mL).

The combination of colistin with Alg NPs, at the MIC value, has a significant effect on the membrane leakage of proteins compared to Alg NPs (without colistin) and colistin alone at the MIC value (Figure 1).

We observed a progressive increase in protein loss compared to Alg NPs (without colistin) and colistin. Similarly, Alg NPs+Colistin also accelerated protein leakage across the membrane of *E. coli* 184 in comparison with colistin alone. Initially, protein leakage from cells in the control experiment (only Alg NPs) was 6.63 µg/mL, whereas protein leakage after treatment with colistin (8 µg/mL) and Alg NPs+Colistin (500:60 µg/mL) was 29.98 and 38.05 µg/mL, respectively.

Protein leakage from cells treated with colistin increased to 77.55 µg/mL after 5 h of incubation, compared to 96.86 µg/mL after 5 h of incubation recorded for Alg NPs+Colistin formulation. Protein leakage upon interaction with the Alg NPs+Colistin was 72.94 µg/mL higher than that of Alg NPs (without colistin) and 19.31 µg/mL higher than that of colistin. The Alg NPs+Colistin formulation exhibited a significant effect on protein leakage compared to colistin and Alg NPs taken separately. After 5 h of incubation, Alg NPs+Colistin increased the protein leakage by up to 305% compared to Alg NPs (without colistin) and 25% compared to colistin (without Alg NPs). Thus, the percentage of protein leakage was maximum upon exposure to Alg NPs+Colistin compared to Alg NPs and colistin. The Alg NPs+Colistin formulation resulted in higher protein loss than colistin solution without Alg NPs, indicating a good antibacterial activity of colistin combined with nanoparticles. In a study by Dixon and Chopra, polymyxin B (200 µg/mL), a molecule very close to colistin, caused the release of 100 µg/mL of intracellular proteins after 1 min, reaching 180 µg/mL after 45 min in *E. coli* [57]. Polymyxin B induced immediate release of periplasmic proteins, including beta-lactamases, and cytoplasmic protein responsible for tetracycline efflux within 5 min of exposure [57]. After 60 min of exposure to polymyxin, a concomitant release of 68% of intracellular proteins (such as beta-galactosidase, pyrophosphatase, and aldolase) was observed.

### 2.6. Determination of the Bacteriostatic or Bactericidal Effect of Colistin in the Different Formulations

To define the bacteriostatic or bactericidal character of colistin, it is necessary to calculate the ratio between the minimum bactericidal concentration (MBC) and the minimum inhibitory concentration (MIC) (R = MBC/MIC). The results obtained are summarized in Table 5.

The results revealed that the MIC and MBC values of the colistin solution (64 µg/mL) were equivalent, while the MBC of the Alg NPs+Colistin (5 µg/mL) was four-fold higher than the MIC (1.25 µg/mL). The MBC values obtained with the other formulations (menthol and lactic acid) were two times higher than the corresponding MIC values. Different studies assessed the value of the MBC/MIC ratio to distinguish bactericidal from bacteriostatic antibiotics [58]. If MBC/MIC ≤ 2, the effect is bactericidal, whereas if MBC/MIC is very far from the MIC value, then the effect is bacteriostatic. Other references suggested that if MBC/MIC = 4, the antibiotic is bactericidal; if MBC/MIC = 8–16, the antibiotic is bacteriostatic; and if MBC/MIC = 32, the bacterium is tolerant to the antibiotic [58]. Based on the first hypothesis, MBC/MIC ≤ 2, we can postulate that the tested formulations, except Alg NPs+Colistin, had a bactericidal effect, and the latter displayed a bacteriostatic effect. According to the second hypothesis, all antibacterial formulations exhibited a bactericidal effect (MBC/MIC = 4).

### 2.7. Killing Curve Assays

**Figure 2:**
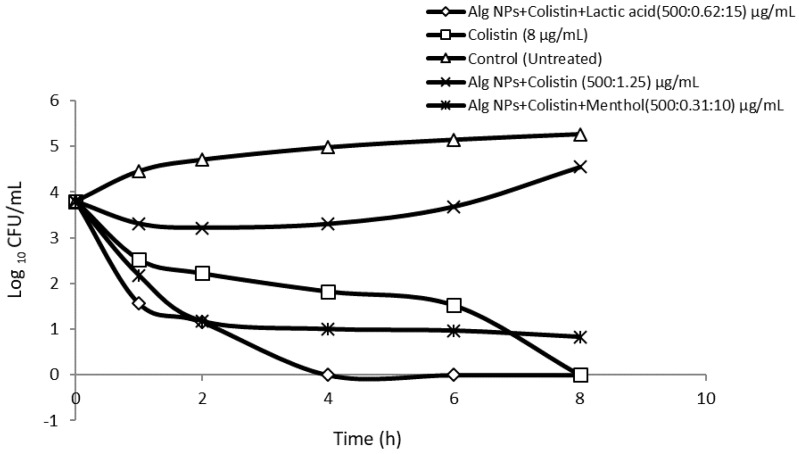
Killing curves of *Escherichia coli* 184 treated with different formulations of colistin associated with Alg NPs and menthol or lactic acid.

To evaluate the antibacterial effects of colistin loaded on Alg NPs in the presence of menthol or lactic acid on *E. coli* 184 (*mcr*-1) strain, measurement of the reduction in CFU/mL within 8 h was performed. As shown in Figure 2, colistin (8 µg/mL) is bactericidal, preventing the regrowth of *E. coli* 184 strain with a continuous decrease of the bacteria population, until reaching an undetectable level within 8 h of incubation. In contrast, Alg NPs+Colistin (500:1.25 µg/mL) reduced the *E. coli* 184 population from 4 log_10_ CFU/mL to 3.2 log_10_ CFU/mL, but regrowth was observed after 4 h. In addition, Alg NPs+Colistin+Menthol and Alg NPs+Colistin+Lactic acid combinations enhanced the bactericidal effect of Alg NPs+Colistin, resulting in a substantial improvement in bacterial killing to 1.1 log_10_ CFU/mL after 2 h. Thus, the combination of Alg NPs+Colistin+Lactic acid was the most effective combination among the three formulations tested, as it resulted in a reduction of the number of bacteria after 4 h of incubation to a level of <0.5 log_10_ CFU/mL, compared to that with colistin (>1.8 log_10_ CFU/mL) and with menthol (1 log_10_ CFU/mL). Thus, the combination of colistin with Alg NPs and lactic acid or menthol had a clearly bactericidal effect.

Colistin features a rapid bactericidal activity related to its permeabilizing action on the cell membrane, following absorption and electrostatic interactions with the bacterial membrane [59]. Of note, it is well-established that its association with certain components of essential oils, characterized by their bactericidal activities against *E. coli* strains, allows a potentiation of its rapid destructive action during the first hour of exposure to such a combination [60].

### 2.8. Transmission Electron Microscopy (TEM) Studies

**Figure 3:**
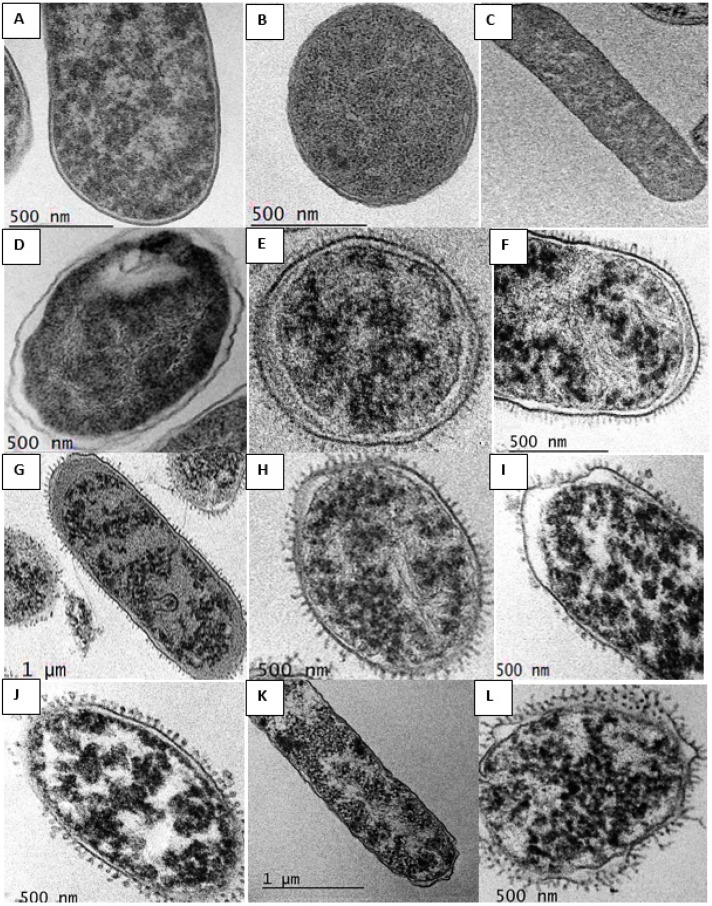
TEM micrographs of *Escherichia coli* 184 cells treated with (**A**–**C**) control; (**D**) Alg NPs (500 µg/mL); (**E**–**F**) Colistin (8 µg/mL); (**G**–**H**) Alg NPs+Colistin (500:1.25) µg/mL; (**I**–**J**) Alg NPs+Colistin+Lactic acid (500:0.62:15) µg/mL; (**K**–**L**) Alg NPs+Colistin+Menthol (500:0.31:10) µg/mL.

TEM was used to study the membrane integrity and possible intracellular alterations of *E. coli* 184 strain cells before and after treatment with the different antibacterial formulations designed in this study. TEM observation of *E. coli* 184 treated with colistin loaded onto Alg NPs in the presence of lactic acid or menthol showed morphological changes on the surface and inside the bacteria that were absent in untreated bacteria (Figure 3).

### 2.9. Stability of Alg NPs-Colistin-Small Molecules in Conditions Mimicking the Passage through the Gastrointestinal Tract

We have previously mentioned the importance of maintaining good colloidal stability of the nanoparticles during antimicrobial activity tests. However, this consideration was very rarely addressed in the tests described in the literature. Hence, we investigated the effect of digestive enzymes on the activity of Alg NPs+Colistin with lactic acid (1 µg/mL) or menthol (10 µg/mL) on *E. coli* 184 over two incubation times (30 min and 2 h) at two pH values (3 and 6). Three enzymes were assessed: pepsin (digestive enzyme of the stomach, active at pH 2–3), trypsin, and chymotrypsin (enzymes synthesized in the pancreas and secreted in the small intestine, active between pH 6 and 8). Table 6 groups the MIC values of the various formulations following the different enzymatic treatments. For the colistin solution (60 µg/mL), the MIC for the control without enzymes and without incubation, and the MIC for the control that underwent incubation at 39 °C for 30 min at pH 3 and then 2 h at pH 6, was 8 µg/mL. In addition, the MIC value for the colistin solution adsorbed on Alg NPs remained at 1.25 µg/mL after treatment, which means that the antibacterial activity of colistin was not affected, and its association with Alg NPs conferred a sort of protection that allowed it to retain part of its activity at the physiological temperature of the piglet. However, the MIC values of colistin adsorbed on Alg NPs associated with menthol or lactic acid increased from 0.31 and 0.62 µg/mL to 1.25 µg/mL. This indicates that colistin had lost part of its activity when combined with lactic acid and menthol at acidic pH, despite the fact that lactic acid and menthol had a potentiating effect on bacterial growth inhibition (see combination of colistin with Alg NPs and menthol or lactic acid).

Concerning the treatment with pepsin, the MIC values of the two different formulations (Alg NPs+Colistin and Alg NPs+Colistin+Lactic acid) were higher than the values recorded with the two controls without treatments and without enzymes. These were increased by four and two times, respectively, indicating that pepsin, an enzyme involved in digestion in the stomach, partially degraded the peptide chain and caused a decrease in the antibacterial activity of the different solutions tested. On the contrary, the MICs of the Alg NPs+Colistin, Alg NPs+Colistin+Menthol, and Alg NPs+Colistin+Lactic acid remained constant (5 and 2.5 µg/mL, respectively), even after addition of the two pancreatic enzymes, which contrasts with the activity of the colistin solution, whose MIC increased by a factor of four (from 8 to 32 µg/mL). Only a small amount of published data exist on the effect of bacterial intestinal infection in pigs on the intestinal absorption of colistin. One study by Rhouma et al., regarding the gastric stability and oral bioavailability of colistin sulfate in pigs exposed to *E. coli* O149, demonstrated the rapid degradation of colistin sulfate following the addition of pepsin [61]. However, the antimicrobial activity of colistin sulfate and its degradation products were not accompanied by a loss, but rather a slight increase in antimicrobial activity, although this increase was statistically insignificant, and no difference was found for MICs [61]. Thus, pepsin did not affect the activity of colistin alone, which is in agreement with our results. Colistin is also resistant to trypsin (pH 4.4 to 7.5), pancreatin (pH 4.4 to 7.5), and erepsin (pH 6.1 to 7.8), but was inactivated by lipase [62]. No information regarding the possible degradation of colistin in the porcine gastrointestinal tract by the enzyme chymotrypsin is available in the literature. According to Falagas and Kasiakou, colistin sulfate and sodium colistimethate are not absorbed from the gastrointestinal tract during oral administration [8]. Solutions of colistin salts are relatively stable at pH 2–6, but become unstable at pH > 6, suggesting that when colistin passes through the gastrointestinal tract, its chemical composition and antibacterial activity are not influenced by the gastric environment.

### 2.10. Cytotoxicity Assays

#### 2.10.1. Alg NPs+Colistin Formulations

The neurological and nephrological toxicity of colistin led to its restricted use from the late 1960s. The nephrotoxicity of colistin is mainly manifested by acute tubular nephropathy, causing a decrease in creatinine clearance, whereas neurological toxicity is manifested at high doses by paresthesias of the extremities and perioral area, fatigue, dizziness, visual disturbances, confusional syndrome, and neuromuscular blockade, which may lead to acute respiratory failure and even apnea [63]. As reported in the study by Naghmouchi et al., colistin displayed 100% toxicity to Vero cells at a concentration of 1000 µg/mL [64]. In contrast, Severino et al. found that encapsulation of polymyxin in solid lipid nanoparticles decreased its nephrotoxicity [65,66]. The cytotoxicity of the different formulations was therefore tested on a human (HT-29) cell line.

**Figure 4:**
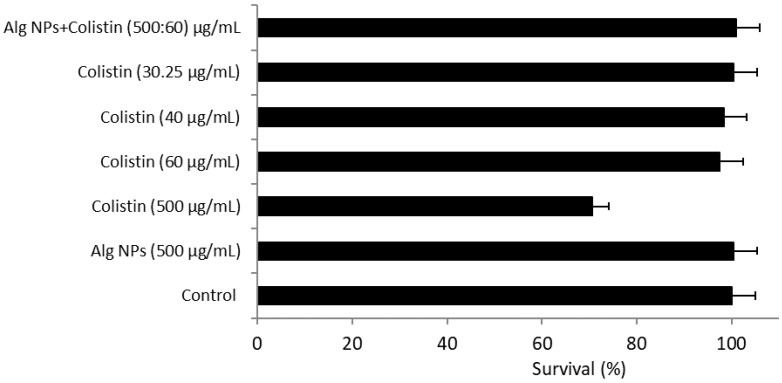
Cytotoxicity of the different colistin formulations on HT-29 cells.

We noted that Alg NPs (500 µg/mL) were completely devoid of any cytotoxic effect, with a percentage of cell survival of approximately 100%. Similarly, the different combinations with Alg NPs were not toxic to HT-29 cells (Figure 4). Among the five tested formulations containing colistin, the colistin solution at a concentration of 500 µg/mL induced a decrease in the HT-29 cells survival (70.5%), in comparison to the other formulations tested, for which the percentage of mortality was lower than 3% (Figure 4).

#### 2.10.2. Alg NPs+Colistin+Components of Essential Oil Formulations

**Figure 5:**
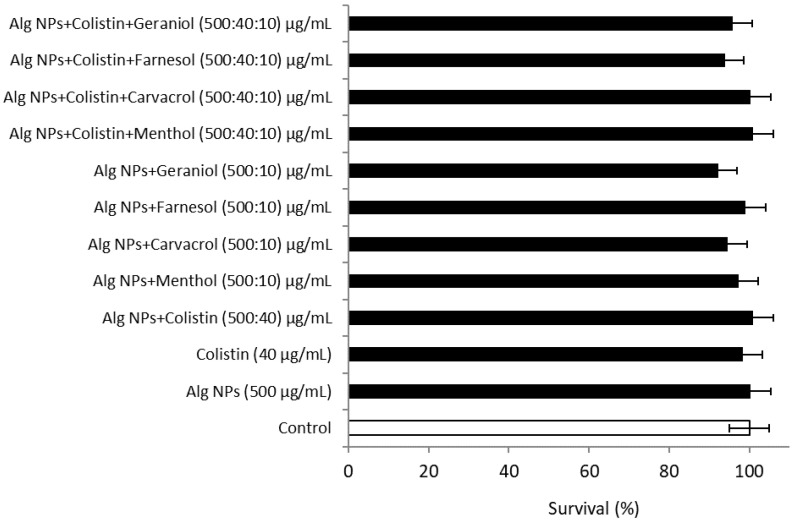
Cytotoxicity of colistin, Alg NPs, and components of essential oil formulations on HT-29 cells.

According to the literature, components of essential oils are increasingly used in therapeutic care, and several published studies have concluded that some components of essential oils may have, under certain conditions, antibacterial, antifungal, or virucidal properties [33]. Despite these properties, components of essential oils could exhibit some toxicity [67]. According to the results obtained with the HT-29 line (Figure S1), the concentration that did not present a toxic effect was 10 µg/mL. It was thus retained for the continuation of the tests carried out with the components of essential oils. The cytotoxicity test of the different combinations of Alg NPs+Colistin with the different components of essential oils was also performed on the HT-29 cell line (Figure 5).

The solutions of the different combinations had a survival percentage higher than 92%. The percentage of survival of Alg NPs+Components of essential oil (menthol, carvacrol, farnesol, or geraniol) without colistin was 92–97%, while the percentage of survival of the formulation containing colistin was 93–100%. This means that the addition of components of essential oils at a concentration of 10 µg/mL did not induce any toxic effect on the HT-29 cell line. According to the results of Figure 5 and those obtained above (cf. test of the cytotoxicity of components of essential oils), the different combinations of Alg NPs with colistin and components of essential oils did not present a toxic effect for the HT-29 cell line.

#### 2.10.3. Alg NPs+Colistin+Lactic Acid

**Figure 6:**
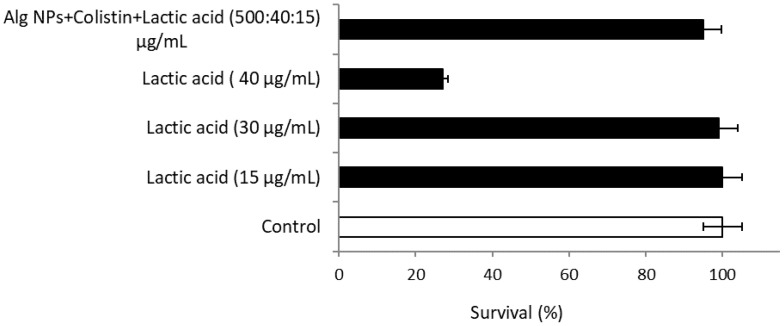
Cytotoxicity of lactic acid and Alg NPs+Colistin+Lactic acid formulation on HT-29 cells.

The percentage of survival of Alg NPs+colistin+lactic acid was 95% for the HT-29 cells (Figure 6). The percentage of survival of HT-29 cells treated with lactic acid solution (40 µg/mL) decreased from 100 to 27%, while at 15 µg/mL, it was about 100%. This indicates that lactic acid at a concentration of 15 µg/mL was not toxic for the HT-29 cell line.

#### 2.10.4. Alg NPs+Colistin+Spermine Formulation

**Figure 7:**
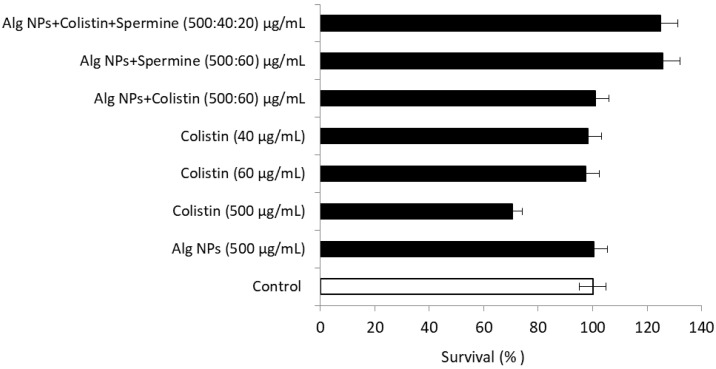
Cytotoxicity of colistin, Alg NPs, and spermine and their combinations on HT-29 cells.

Polyamines are found in the majority of animal and plant cells, and are essential components for cell functions and division, with targets ranging from nucleic acids to nutrient lipids [68]. As noted earlier, the major (and most studied) polyamines are putrescine, spermidine, and spermine. In order to confirm the safety of the two spermine solutions studied above, a cytotoxicity test was performed on the HT-29 cell line (Figure 7).

The percentage of survival of HT-29 cells treated with the two formulations comprising spermine was greater than 100% (126% for Alg NPs+Spermine and 125% for Alg NPs+Colistin+Spermine). A comparison of the percentage of survival of Alg NPs+Colistin without spermine and Alg NPs+Spermine without colistin revealed a percentage of survival of 125% with spermine, whereas with colistin, it was 101%. Thus, spermine, instead of altering cell survival, stimulates the metabolic activity of HT-29 cells and is therefore devoid of toxic effect at the investigated concentrations. The majority of previous studies showed that polyamines (spermine and spermidine) are an essential class of metabolites present in all kingdoms of the living world and are factors in cell growth and proliferation [68]. On the other hand, other studies highlighted that these polyamines exhibit acute and subacute toxicity in rats. Spermidine and spermine were both acutely orally toxic at 600 mg/Kg body weight. Spermine was most toxic, at 200 ppm (19 mg/Kg) [69].

## 3. Experimental Section

### 3.1. Escherichia coli (E. coli) Strains Used in This Study

The *E. coli* strains used here are listed in Table 7.

### 3.2. Colistin Calibration Curve

A stock solution of colistin (5 mg/mL) was prepared by dissolving colistin sulfate salt powder (Sigma-Aldrich, St. Quentin Fallavier, France) in distilled water. The solution was diluted in an appropriate volume of diluent (distilled sterile water, brain heart infusion (BHI) broth (Sigma-Aldrich, St. Quentin Fallavier, France) or Dulbecco’s Modified Eagle Medium (DMEM) (Gibco^TM^, Thermo Fisher Scientific, Waltham, MA, USA)) to a working concentration of 500 µg/mL. A series of solutions of colistin of different concentrations (2, 5, 10, 20, 30, 50, 75, 100, 125, 150, 175, 200, 225, 250, and 500 µg/mL) were prepared by dilution in water and homogenized by sonication for 15 min at 25 °C.

### 3.3. Formation of Alginate Nanoparticles (Alg NPs)

The formation of the Alg NPs was carried out by a top-down process by ball milling using planetary mixer PM100 (Retsch GmbH, Haan, Germany) and characterized by scanning electron microscopy and zeta potential measurements, according to our recently published reports [70,71]. In short, 2 g of alginic acid sodium salt (Sigma-Aldrich, St. Quentin Fallavier, France) was placed in a crushing pot (1.050 kg) for 10 h at 440 rpm in contact with 112 g of zirconium oxide beads (3 balls of 20 mm diameter and 10 balls of 10 mm diameter) (Retsch GmbH, Haan, Germany). At the end of the grinding process, homogeneous suspensions of nanoparticles (500 µg/mL) in water were prepared by ultrasonication for 60 min at 25 °C.

### 3.4. Preparation of Colistin-Loaded Alg NPs and Quantification

Suspensions of Alg NPs+Colistin were prepared at different colistin concentrations, according to well-defined steps. A constant Alg NPs concentration of 500 μg/mL was used in order to avoid precipitation and ensure appropriate dispersibility. This solution was mixed with different solutions of colistin previously prepared and homogenized by sonication for 60 min at 25 °C. The amount of colistin was determined by reverse-phase high-performance liquid chromatography (RP-HPLC). RP-HPLC analyses were performed on a Shimadzu LC2010-HT apparatus (Shimadzu, Tokyo, Japan). A column (C4 QS Uptisphere^®^ 300 Å, 250 × 4.6 mm (Interchim, Montluçon, France)) of 5 mm was used as the analysis column. The column was heated to 40 °C. The mobile phase was a mixture of eluent A (0.1% trifluoroacetic acid in H_2_O) and eluent B (0.09% trifluoroacetic acid, 80% CH_3_CN, and 19.91% H_2_O) at a flow rate of 1 mL / min. The linear gradient was 0–80% of eluent B for 30 min, and the detection was recorded at 215 nm. All experiments were performed at room temperature, and the total area of the colistin peak was used for its quantification.

Suspensions of Alg NPs+Colistin were prepared at different percentage ratios of colistin to Alg NPs from 0% to 20%. The resulting Alg NPs+Colistin suspensions were then dialyzed for 24 h at room temperature. These solutions were injected into the HPLC column, and colistin elution during the time of the experiment was monitored with a UV detector at 215 nm. Concentrations of 25 to 500 μg/mL of colistin prepared in water were used to generate a calibration curve for the calculation of the colistin concentration (in μg/mL) relative to the peak area obtained.

### 3.5. Preparation of Alg NPs Loaded with Colistin and Small Molecules (Components of Essential Oils, Polyamines, Organic Acids) Formulations

Formulations of Alg NPs+Colistin+Small molecules were prepared at different concentrations, according to well-defined steps. In each solution, the concentration of the Alg NPs was 500 µg/mL.

- Alg NPs+Colistin+Components of essential oil (components of essential oils = menthol, farnesol, geraniol or carvacrol) (Sigma-Aldrich, St. Quentin Fallavier, France) formulations at different concentrations of colistin (30, 40 or 50) µg/mL and fixed concentration of component of essential oils (10 μg/mL), chosen on the light of preliminary cytotoxicity assay (Figure S1).
- Alg NPs+Colistin+Polyamines (spermine or piperazine) (Sigma-Aldrich, St. Quentin Fallavier, France) formulations: colistin = 40 µg/mL and polyamine = 20 μg/mL.
- Alg NPs+Colistin+Spermidine (Sigma-Aldrich, St. Quentin Fallavier, France) formulations: colistin = 30, 20, or 10 µg/mL and spermidine = 30, 40, and 50 µg/mL.
- Alg NPs+Colistin+Lactic acid (Sigma-Aldrich, St. Quentin Fallavier, France) formulation: colistin = 40 μg/mL and lactic acid = 15 μg/mL.

All formulations were finally homogenized by sonication for 60 min at 25 °C.

### 3.6. Determination of Minimal Inhibitory Concentration (MIC) and Minimal Bactericidal Concentrations (MBC)

Minimal inhibitory concentration (MIC) determination of the aforementioned formulations was carried out in triplicate in 96-well microtiter plates using the broth microdilution method described in the Clinical and Laboratory Standards Institute guidelines (CLSI, Wayne, NJ, USA (M27-A2)) [72,73]. Fresh colonies of *E. coli* were sub-cultured in Mueller-Hinton broth at 37 °C on a rotary shaker overnight at 160 rpm. MICs were determined in 96-well round-bottom plates using a volume of 250 μL inoculated with 1% (*v*/*v*) of the *E. coli* strain sub-culture. The two-fold serial dilution concentrations of colistin ranged from 40 to 0.312 µg/mL. The plates were incubated for 24 h at 37 °C, and inhibition of growth was confirmed visually and turbidimetrically.

Minimal bactericidal concentration (MBC) is the lowest concentration of antibiotic that leaves only 0.01% or less of viable cells of the initial inoculum after 18 h incubation at 37 °C, and it characterizes the bactericidal effect of an antibiotic. MBC was determined from the MIC; the method used consists of sub-culturing, on a plate of BHI agar medium, of 100 µL of *E. coli* 184 (*mcr*-1) strain from a broth culture containing the MIC of the antibiotic. Viable cells were then evaluated after 24 h of incubation at 37 °C by the colony count method [74].

MBC was determined for colistin (60 µg/mL), Alg NPs+Colistin (500:60 µg/mL), Alg NPs+Colistin+Menthol (500:40:10 µg/mL), and Alg NPs+Colistin+Lactic acid (500:40:15 µg/mL). The number of viable cells after treatment was compared to the number of bacteria in the initial inoculum, which was also evaluated by colony counting.

### 3.7. Effect of Alg NPs and Alg NPs+Colistin on Leakage of Proteins on E. coli 184 (mcr-1) Strain

First, 150 µL of Alg NPs and Alg NPs+Colistin was added to 50 mL of BHI broth containing 7 Log _10_ CFU/mL of bacterial culture and incubated at 37 ± 2 °C with continuous shaking at 200 rpm. Then, 1 mL of the treated bacterial culture was collected at every 2 h interval up to 8 h. Samples were centrifuged at 12,000 rpm for 5 min, and the supernatants were immediately frozen at −20 °C. The concentration of proteins from each sample was determined immediately using QuantiPro BCA assay kit (Sigma-Aldrich, St. Quentin Fallavier, France). A bacterial culture with colistin suspension at MIC was used as control.

### 3.8. Killing Curves

Time-kill curve studies were performed on a colistin-resistant isolate (*E. coli* 184). Colistin, Alg NPs+Colistin or Alg NPs+Colistin+Small molecules, at previously determined MIC, were added to a log-phase inoculum of roughly 7 Log _10_ CFU/mL to a final volume of 10 mL of BHI broth (Sigma Aldrich, St. Quentin Fallavier, France) culture prepared on the day of the experiment. Inoculated broths were then incubated at 37 °C, and then 1 mL of inoculated broth was sampled at different time intervals (0, 2, 4, 8, 12, 24, and 36 h) from each tube and subjected to 10-fold serial dilutions. Then, 0.1 mL of every dilution was spread on Mueller–Hinton agar plates (Sigma Aldrich, St. Quentin Fallavier, France) and incubated at 37 °C for 24 h. Then, visible *E. coli* colonies on plates were counted, allowing the drawing of the time-kill curves [75].

### 3.9. Transmission Electron Microscopy (TEM)

Transmission electron microscopy (TEM) images were acquired using a JEOL JEM-2100 (JEOL, Tokyo, Japan) transmission electron microscope operating at an acceleration voltage of 200 KV. After determination of the MIC of different formulations (colistin (60 μg/mL), Alg NPs (500 μg/mL), Alg NPs+Colistin (500:40 μg/mL), Alg NPs+Colistin+Menthol (500:40:10 µg/mL), Alg NPs+Colistin+Lactic acid (500:40:15 µg/mL)), the suspensions of the wells where there was an inhibition of bacterial growth were taken in Eppendorf tubes, sedimented by centrifugation (9000 rpm, 60 min, 4 °C), and washed twice with phosphate-buffered solution (PBS). The samples were fixed with 2.5% (*v*/*v*) and 2% (*v*/*v*) glutaraldehyde in 0.1 M PBS containing 0.5 mM MgCl_2_ (pH 6.5), dehydrated and included in EPON resin (TAAB, Berks, England). Ultrafine sections (40 to 60 nm thick) were prepared with an RMC ultramicrotome (Power Tomes, Boeckeler Instruments Inc, Tucson, AZ, USA) using a diamond knife (DDK, Wilmington, DE, USA). The sections were mounted on 200 mesh nickel or copper grids, stained with 2% (*w*/*v*) uranyl acetate and 3% (*w*/*v*) lead citrate for 5 min, and examined under TEM JEOL JEM-2100 (JEOL, Tokyo, Japan). Images were digitally recorded using a CCD digital camera Orious 1100 W (Tokyo, Japan).

### 3.10. Evaluation of Alg NPs-Colistin-Small Molecules Formulations Stability in the Conditions Mimicking the Passage through the Gastrointestinal Tract 

To assess the stability of the previously designed Alg NPs-Colistin formulations during their passage throughout the harsh conditions of a simulated gastrointestinal (GIT) we proceeded according to the protocol described by Zgheib et al. [22] with some modifications. Briefly the gastric compartment was obtained using a solution containing 15U/mL of pepsin (Sigma Aldrich, St Quentin Fallavier, France) in PBS adjusted at pH 3 with 0.5M HCl, incubated at 39°C with continuous agitation (160 rpm) for 30 min, followed by a simulated duodenal compartment solution with addition of 40U/µL of trypsin and 5U/mL of chymotrypsin, both obtained from Sigma-Aldrich (St Quentin Fallavier, France) after pH adjustment to 6 and a further incubation of 2 h under the same conditions. The formulations were also subjected to the same treatment without the trypsin and chymotrypsin in one case and without the three enzymes in another case. The MIC of the colistin was then determined for all the formulations after the treatment as described above.

### 3.11. Cytotoxicity Assay

The cytotoxicity assay was performed using an adapted protocol described by Belguesmia et al. [76]. Briefly, human colon carcinoma (HT-29) cells (Sigma-Aldrich, St. Quentin Fallavier, France) were cultivated in 96-well tissue culture plates for 48–72 h at 37 °C, in atmosphere containing 5% CO_2_, until the formation of a continuous confluent cell culture on the bottom of each well. The Alg NPs+Colistin and Alg NPs+Colistin+Small molecules were tested at their previously determined MIC values, as described above. The required concentrations were prepared in DMEM without antibiotics and serum and were added to the HT-29 cells in the wells, after washing with the same medium without colistin formulation. Then, the treated HT-29 cells were incubated for 24 h at 37 °C, in atmosphere containing 5% CO_2_. CCK8 assay (Dojindo Molecular Technologies Inc, Rockville, MD, USA), based on the reduction of tetrazolium salt by active mitochondria, was used to assess cell viability of the treated HT-29 cells. For this, 150 µL of DMEM containing 7.5 µL of CCK-8 reagents was added in each well, and cells were incubated for 2 h. Plates were read at 450 nm in a microplate reader spectrophotometer (Xenius, Safas, Monaco). Results were expressed as % of basal growth observed with non-treated cells.

## 4. Conclusions

Colistin loaded on alginate nanoparticles (Alg NPs) enhanced its antibacterial activity by decreasing its MIC value from 8 to 1.25 µg/mL against *E. coli* 184. This strain carries the *mcr*-1 gene, which is involved in colistin resistance. Furthermore, simultaneously loading colistin and small molecules such as components of essential oils (menthol, carvacrol, farnesol, or geraniol), or lactic acid on Alg NPs improved its potency. Indeed, MIC values of 0.31 or 0.62 µg/mL were recorded when colistin with menthol or lactic acid were co-loaded on Alg NPs. The formulations designed in study conferred better stability to colistin under simulated GIT conditions, accompanied with a very limited cytotoxicity on HT-29 intestinal cells. Taken together, this new approach based on the combination of antibiotic, nanoparticles, and small molecules represents a promising prospect to treat infections ascribed to Gram-negative bacilli, particularly those related to resistance to colistin. Assessment of these novel and promising formulations in vivo constitutes our next goal.

## Figures and Tables

**Table 1:** MIC of colistin alone and in combination with Alg NPs against different *E. coli* strains.

Strains	Colistin (MIC [µg/mL])	Alg NPs+Colistin (MIC [µg/mL])
*E. coli* 184	8	1
*E. coli* 289	8	4
*E. coli* E4A4v	16	4
*E. coli* E5A16v	16	4
*E. coli* ATTC8739	2	1
*E. coli* CIP 7624	1	1
*E. coli* SBS363	2	1
*E. coli* E4A4wt	2	2
*E. coli* E5A16wt	2	2
*E. coli* Top 10	1	0.5

**Table 2:** MIC of colistin (in µg/mL) alone and in combination with Alg NPs and components of essential oils against *Escherichia coli* 184 strain.

Formulations (µg/mL)	MIC (µg/mL)
Alg NPs+Menthol (500:10)	-
Alg NPs+Carvacrol (500:10)	-
Alg NPs+Farnesol (500:10)	-
Alg NPs+Geraniol (500:10)	-
Alg NPs+Colistin+Menthol (500:40:10)	0.31
Alg NPs+Colistin+Carvacrol (500:40:10)	≤0.62
Alg NPs+Colistin+Farnesol (500:40:10)	≤1.25
Alg NPs+Colistin+Geraniol (500:40:10)	≤1.25

-: No inhibition.

**Table 3:** MIC of colistin (in µg/mL), alone and in combination with Alg NPs and lactic acid, against *Escherichia coli* 184 strain.

Formulation (µg/mL)	MIC (µg/mL)
Lactic acid (15)	-
Alg NPs+Colistin (500:40)	1.25
Alg NPs+Colistin+Lactic acid (500:40:15)	≤0.62

-: No inhibition.

**Table 4:** MIC of colistin (in µg/mL), alone and in combination with Alg NPs and polyamines, against *Escherichia coli* 184 strain.

Formulation (µg/mL)	MIC (µg/mL)
Colistin	8
ACS Alg NPs+Colistin+Spermine (500:60:20)	5
ACSd: Alg NPs+Colistin+Spermidine (500:60:20)	2.5
ACP: Alg NPs+Colistin+Piperazine (500:60:20)	10
ACSd_1_: Alg NPs+Colistin+Spermidine (500:30:30)	3.75
ACSd_2_: Alg NPs+Colistin+Spermidine (500:20:40)	5
ACSd_3_: Alg NPs+Colistin+Spermidine (500:10:50)	5

**Table 5:** MIC *vs.* MBC values of colistin in the different formulations tested against *Escherichia coli* 184.

Formulation	MIC (µg/mL)	MBC (µg/mL)	MBC/MIC
Colistin	8	8	1
Alg NPs+Colistin (500:40)	1.25	5	4
Alg NPs+Colistin+Menthol (500:40:10)	0.31	0.62	2
Alg NPs+Colistin+Lactic acid (500:40:15)	≤0.62	1.25	2

**Table 6:** MIC values of colistin (µg/mL), alone and upon association with Alg NPs and lactic acid or menthol, on *Escherichia coli* 184 (*mcr*-1) strain after treatment mimicking the passage through the gastrointestinal tract.

Formulation	Untreated	Incubation at pH 3 for 30 min and then at pH 6 for 2 h	Incubation at pH 3 for 30 min with Pepsin * and then at pH 6 for 2 h	Incubation at pH 3 for 30 min with Pepsin * and then at pH 6 for 2 h with Trypsin+Chymotrypsin *
Colistin (60 µg/mL)	8	8	8	≥32
Alg NPs+Colistin (500:40) µg/mL)	1.25	1.25	5	5
Alg NPs+Colistin+Lactic acid (500:40:10 µg/mL)	0.62	1.25	2.5	2.5
Alg NPs+Colistin+Menthol (500:40:10) µg/mL	0.31	1.25	1.25	2.5

* Pepsin: 15 U/mL; trypsin: 40 U/µL; chymotrypsin: 5 U/mL.

**Table 7:** *E. coli* strains used in this study.

Strains	Origin	Characteristics
*E. coli* 184	Résapath network *,	Colistin ^R^, (*mcr*-1 gene)
*E. coli* 289	ANSES ** collection,	Colistin ^R^, (*mcr*-4 gene)
*E. coli* E4A4wt	ANSES collection	Colistin ^S^
*E. coli* E5A16wt	ANSES collection	Colistin ^S^
*E. coli* ATTC8739	ATCC collection	Colistin ^S^, reference strain
*E. coli* CIP 7624	Pasteur Institute collection (Paris, France)	Colistin ^S^, reference strain
*E. coli* SBS363	Pasteur Institute collection (Paris, France)	Strain with truncated LPS
*E. coli* E4A4v	ANSES collection	In vitro resistant variant to colistin
*E. coli* E5A16v	ANSES collection	In vitro resistant variant to colistin
*E. coli* Top 10	Invitrogen ^®^	Modified strain (LPS^-^)

* French Résapath network for the surveillance of antimicrobial resistance in pathogenic bacteria of animal origin: https//www.resapath.anses.fr/ (accessed 23 May 2022). ** ANSES: French agency for food, environmental, and occupational health and safety. Colistin ^R^: resistance to colistin (minimal inhibitory concentration (MIC) > 2 mg/L). Colistin ^S^: susceptible to colistin (MIC ≤ 2 mg/L).

## Data Availability

Data is contained within the article and supplementary material.

## Supplementary Materials

The following supporting information can be downloaded at: https://www.mdpi.com/article/10.3390/ph15060682/s1, Figure S1: Cytotoxicity of components of essential oils on HT-29 cells.
