# Supplementary material for: Enhancing Colistin Activity against Colistin-Resistant Escherichia coli through Combination with Alginate Nanoparticles and Small Molecules
Source: Pharmaceuticals (Basel). 2022 May 28;15(6):682. doi: 10.3390/ph15060682 (PMC9227550; doi:10.3390/ph15060682)
Supplement: Supplementary file 1 [file pharmaceuticals-15-00682-s001.zip › pharmaceuticals-1707327-supplementary.pdf]

Supplementary information

Enhancing colistin activity against colistin-resistant *Escherichia coli* through combination with alginate nanoparticles and small molecules

Noura Hazime [1,2,†], Yanath Belguesmia [2,†], Isabelle Kempf [3], Alexandre Barras [1], Djamel Drider [2] and Rabah Boukherroub [1,*]

[1] Univ. Lille, CNRS, Centrale Lille, Univ. Polytechnique Hauts-de-France, UMR 8520, IEMN, F-59000 Lille, France; noura.hazime@hotmail.fr (N.H.); alexandre.barras@univ-lille.fr (A.B.)
[2] UMR Transfrontalière BioEcoAgro1158, Univ. Lille, INRAE, Univ. Liège, UPJV, YNCREA, Univ. Artois, Univ. Littoral Côte D'Opale, ICV-Institut Charles Viollette, 59000 Lille, France; yanath.belguesmia@univ-lille.fr (Y.B.); djamel.drider@univ-lille.fr (D.D.)
[3] ANSES, Laboratoire de Ploufragan-Plouzané-Niort, Unité Mycoplasmologie Bactériologie Antibiorésistance, 22440 Ploufragan, France; isabelle.kempf@anses.fr (I.K.)
* Correspondence: rabah.boukherroub@univ-lille.fr
† These authors contributed equally to this work.

Chemicals and reagents

Colistin, alginic acid sodium salt (powder, viscosity: 15-25 cP, 1% in H_2O), colistin, (1R, 2S, 5R), menthol, farnesol (95%), geraniol (98%), carvacrol (98%), lactic acid, spermine and piperazine and spermidine were purchased from Sigma-Aldrich (St Quentin Fallavier, France). Microbiological media, Brain Heart Infusion (BHI), was purchased from Sigma-Aldrich (St Quentin Fallavier, France). Cell culture Dulbecco's Modified Eagle's Medium (DMEM) was purchased from Gibco™ (Thermo Fisher Scientific, Waltham, MA, USA)

Characterization of Alg NPs

The size and zeta potential of Alg NPs were determined at 25°C using a Zetasizer Nano ZS (Malvern Instruments S.A., Worcestershire, UK) in 173° scattering geometry and the zeta potential was measured using the electrophoretic mode. The analysis of the size distribution was performed using the dynamic light scattering method (DLS). Immediately before the measurement, the sample was suspended in sterile water, vortexed for 5 min and sonicated in an ultrasonic bath for 60 min at 25 °C. All measurements were performed in triplicate for each sample using UV-grade cuvettes for size determination and folded capillary cells to record the zeta potential (ZP).

Cytotoxicity test of components of essential oils used in this study

Components of essential oils at a concentration of 10 μg/mL were not cytotoxic for HT-29 cells (viability > 90%). The percentage of cell survival was lower than 70% for components of essential oil concentration of 20 μg/mL, while at a concentration of 40 μg/mL, the percentage of cell alteration was greater than 75%. This means that the maximum amount of components of essential oils that could be used and that does not present a significant toxic effect is 10 μg/mL.

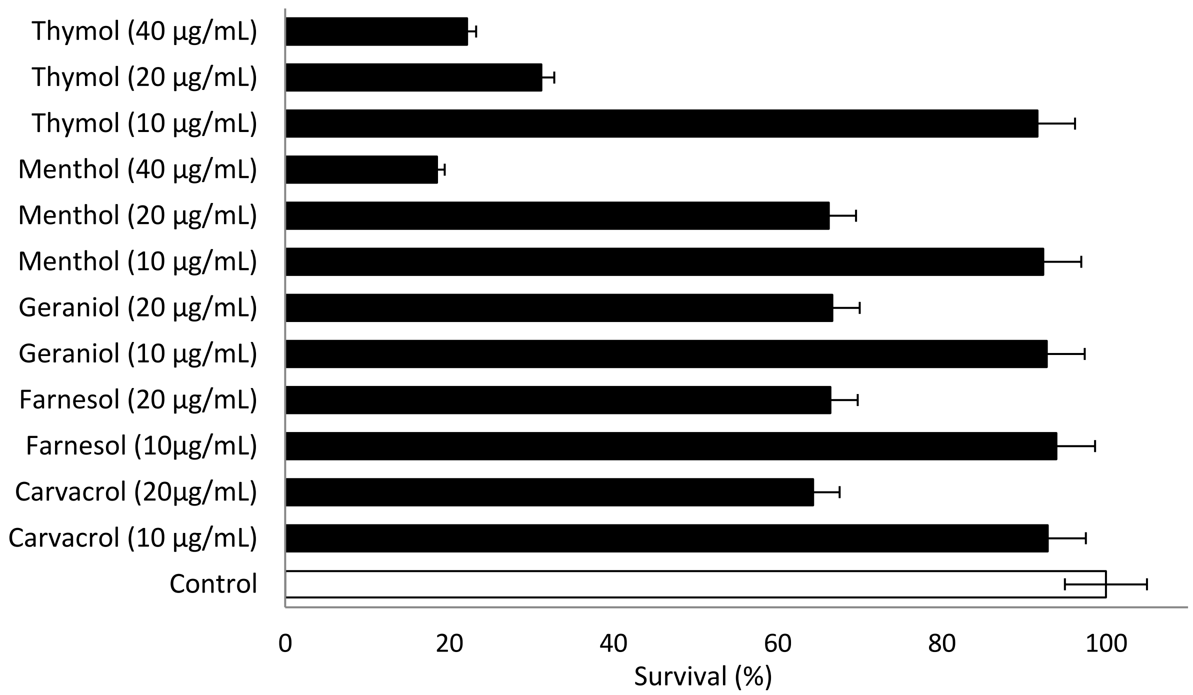

Figure S1. Cytotoxicity of components of essential oils on HT-29 cells.
